# Sex assessment from the pelvis: a test of the Phenice (1969) and Klales et al. (2012) methods

**DOI:** 10.1007/s12024-023-00685-4

**Published:** 2023-08-03

**Authors:** Vanessa Rae Jager, Constantine Eliopoulos

**Affiliations:** James Parsons building, School of Biological and Environmental Sciences, Liverpool John Moores University, Byrom Street, Liverpool, L3 3AF UK

**Keywords:** Forensic anthropology, Sex assessment, Phenice method, Pubic bone

## Abstract

Sex assessment is one of the first steps of routine forensic anthropological examinations and it provides a crucial element to identify a set of human skeletal remains. In bioarchaeological contexts, this assessment is also important, as it helps in the reconstruction of past societies. Sex determination can be achieved by using several morphological or metric traits of the skull and postcranial skeleton, which have been found to have varying degrees of accuracy. In 1969 Phenice proposed a methodology focusing on three traits located on the pubis. These traits were described as either having a female or male morphology with ambiguity being rare. Phenice’s method became regularly utilized as it was considered to be reliable. In 2012, Klales and colleagues published a revision of Phenice’s method, as they found that it did not capture the variation in the expression of the three traits. Klales and co-authors created a visual ordinal scale of 1–5 for each of the three traits Phenice originally identified, thus adding three extra possible forms of expression. The purpose of the present research was to test both the original and revised methodologies on the same skeletal population in order to evaluate their suitability for the assessment of sex. The Luís Lopes Anthropological collection in Lisbon was used; 117 males and 117 females were scored using both methodologies. The results showed that the original method performed better (96.5% accuracy) than the revised method (92.7%).

## Introduction

The pubis has commonly been acknowledged as one of the principle indicators of sex in skeletal remains, due to its high degree of sexual dimorphism [1–7]. The two categories of methodology regularly utilized for sex determination using the innominates include morphological and metric techniques; with the former being preferred for practicality and lack of specialized equipment required [1, 2, 5, 8–12]. However, morphological methods have limitations, such as the subjective nature of some of the traits and the requirement for excellent knowledge of the human skeleton [1, 13].

In 1969, Phenice published a non-metric method for sex determination using visual scoring criteria of drawings and descriptions for three features on the pubis, with a 96% accuracy rate. The method is based on the morphological variation between males and females for three traits; the ventral arc, subpubic concavity and the medial aspect of the ischio-pubic ramus. The original test population consisted of 275 adult skeletons of known sex with both black and white ancestries from the Terry Skeletal Collection. Variation of accuracy rates between the ancestral groups was observed favoring males and females from the white group [2]. However, Phenice’s findings went on to be successfully replicated and utilized in many different studies; albeit most did not reach an accuracy rate as high as the original publication [1, 2, 14–25].

In 2012, Klales and colleagues published a revision on Phenice’s method, with the intention to rectify the lack of intermediate scores by using a comprehensive description for each of the original traits. The rationale of that study was that the additional grades of expression would capture a wider range of variation in the morphology of the traits [1]. Phenice did recognize that further detailed research could result in clarification of the technique, enabling greater accuracy; a clear objective that Klales and colleagues undertook [1, 2]. Improving the original methodology by also creating a quantifiable technique in order for it to be admissible in court under the *Daubert* requirements, which the original method fails to accommodate, was also a priority [26]. The results of the revised method provided a combined accuracy of 86.2%, (98% females, and 74.4% males); when inexperienced scorers were removed, the combined accuracy rose to 94.5% [1]. No significant effects of ancestry were found, allowing for pooling of all ancestral groups for the revised method. Statistical analysis to determine error-rates and frequencies along with a logistical regression equation applied to the trait scores was also established by the revised method authors [1].

Despite the apparent success rate of the revised method, it has yet to be directly compared to the original method using the same modern skeletal population. Most of the recent publications regarding the revised method, are concerned with how the logistic regression equation works for a particular population [1, 27–30]. The primary aim of the current study is instead to focus on the visual scoring system using the 1–5 ordinal scale for each trait from the revised method and compare it to the original methodology in order to determine which technique has the highest accuracy for sex assessment. This is the first study to test both methods on the same skeletal population. A further aim of this study is to ascertain whether there are any sex biases present for females or males in either the revised or the original methods.

## Materials and methods

The skeletal remains used for the present research come from the Luís Lopes Anthropological Collection which is housed at the Natural History Museum in Lisbon, Portugal. This collection consists of 1,692 skeletal remains, all with documented data including sex, age at death, place of birth, occupation, place of residence as well as cause of death. All individuals are of Portuguese ancestry from the late 19th and early to mid-20th centuries whose remains were collected from three Lisbon cemeteries starting in 1981 [31]. For this study, a random stratified sample of 234 individuals of known sex were examined.

The sample was balanced in regards to sex, as 117 males and 117 females were studied. Observations were made on the left pubic bone unless it was damaged or absent and in these cases the right side was used. A total of 27 side substitutions were made, 12 for males and 15 for females. Age at death for individuals ranged from 18–86, with a mean of 61 years. The innominates observed were from adult individuals with an intact pubic bone and with no obvious pathological conditions affecting the pubis.

For this research the ventral arc, subpubic concavity and the medial aspect of the ischiopubic ramus were scored according to the methodology of the respective publication, the original or revised. The final sex assessment for each specimen examined was reached from the majority of features utilized (2 out of 3 traits) [1, 2]. For the purpose of the present study, innominates given scores of either probable female, or probable male, were classified as female or male respectively. The innominates with mixed scores were placed in the indeterminate category.

The primary observer for the study was a postgraduate anthropology student (VRJ) with extensive osteological knowledge and experience with the practical application of both the original and revised methodologies. In order to test the scoring ability of the observer, the intra-observer error was calculated. This was achieved by having the observer rescore 50 innominates from the original sample of 234, which had been chosen at random. These re-scored innominates were then compared to their original scores to check for any discrepancies, which tested the observer’s ability to consistently apply both methods.

While scoring both the original Phenice method (1969) and Klales and colleagues (2012) revised method, a group of 5–10 innominates were scored during each session. Each innominate was scored according to the three traits independently, with reference to each scoring system for the appropriate method. This means while an innominate was being scored using the original Phenice method (1969) the visual aid and descriptions from the publication was being referenced. This was also done when the 2012 revised methodology was being used. Each method was scored independently, with time left in between each method to allow for data entry before carrying on to the next method. This allowed for a non-biased approach during scoring of both methodologies.

To determine whether the original Phenice or the revised method was more accurate at assessing sex, a McNemar’s chi-squared test was used [32]. Two McNemar’s tests were also run, the first to test for intra-observer error and the second to determine whether there was a difference between females and males being sexed incorrectly for the original Phenice method. Finally, a chi-squared test was used to test the differences between males and females for the revised methodology [32]. Sex bias was calculated through the difference between accuracy rates of males and females. Statistics software SPSS 24.0 was used for all statistical analyses [33].

## Results

Intra-observer error was assessed using an exact McNemar’s test for both the revised and original methodologies. For both methods, intra-observer error demonstrated that there was no statistically significant difference between the two observations of the 50 innominates, *(p* > 1.00).

The accuracy of the original and revised methods was assessed for each individual trait, followed by a comparative assessment between the two to determine which methodology provides the best results. The accuracy rates for each of the three traits for the original Phenice method are presented in Table 1 and for the revised methodology by Klales and colleagues in Table 2.

**Table 1:** Accuracy rates for the three pubic traits when using the Phenice methodology for each sex and combined sexes

Sex	Ventral Arc	Subpubic concavity	Ischiopubic ramus
Male	113 (96.58%)	109 (93.16%)	109 (93.16%)
(n = 117)	d		
Female	112 (95.73%)	113 (96.58%)	108 (92.31%)
(n = 117)			
Combined	225 (96.15%)	222 (94.87%)	217 (92.74%)
(n = 234)

**Table 2:** Accuracy rates for the three pubic traits when using the Klales et al. revised methodology for each sex and combined sexes

Sex	Ventral Arc	Subpubic concavity	Ischiopubic ramus
Male	106 (90.16%)	103 (88.03%)	105 (89.74%)
(n = 117)	d		
Female	109 (93.16%)	113 (96.58%)	106 (90.59%)
(n = 117)			
Combined	215 (91.88%)	216 (92.31%)	211 (90.17%)
(n = 234)			

### Ventral arc

The trait that performed best for males was the ventral arc for both the original and revised methodologies, with accuracy rates of 96.58% and 90.16%, respectively. For females, the ventral arc only had a slightly lower accuracy than the males using the original method, 95.73%. For the revised methodology, females actually had a higher accuracy than the males at 93.16%. When both males and females were combined, the original method had a classification accuracy of 96.15%, while the revised method’s accuracy was 91.88%.

### Subpubic concavity

The best performing trait for females was the subpubic concavity as both methodologies used had an accuracy rate of 96.58%. For males using the original methodology, the subpubic concavity had the lowest accuracy at 93.16%. This was also true for males scored with the revised methodology at 88.03%. When both females and males were combined the accuracy rate for the original method was 94.87% and for the revised 92.31%.

### Medial aspect of the ischiopubic ramus

The trait with the lowest accuracy for females was the medial aspect of the ischiopubic ramus, with 92.31% for the original and 90.59% for the revised methodologies, respectively. For the original methodology with males, this trait tied for the lowest classification rate with the subpubic concavity at 93.16%. When the revised methodology was used for males, the medial aspect of the ischiopubic ramus had a relatively low accuracy of 89.74%. When both male and female samples were combined, accuracy reached 92.74% for the original method and 90.17% for the revised methodology which were the lowest accuracy rates among all three traits.

The distribution of the scores on the ordinal scale from the revised method is presented in Table 3. The largest proportion of the sample was given a score of 1, for female, with the second highest given a score of 5, for male. Both scores for probable female and probable male were selected an equal number of times. Score 3, which is classified as ambiguous or indeterminate was used the least of all the scores.

**Table 3:** The distribution of scores using the ordinal scale of the revised Klales et al. method

Sex	Female	Probable female	Ambiguous	Probable male	Male
Score	1	2	3	4	5
n = 234	93	24	7	24	86

In order to compare the original and the revised methods to establish which of the two visual methods is best suited for sex assessment, a McNemar’s chi-squared test with continuity correction was used along with the correct classification rates which can be found in Table 4 for both methods.

**Table 4:** Accuracy rates for both the original and revised methods

Method	Correct	Incorrect	%
Phenice	226	8	96.58%
(n = 234)			
Klales et al. 2012	217	17	92.74%
(n = 234)			

When the original and revised methods were compared using the McNemar’s chi-squared test with continuity correction, there was a statistically significant difference between the two methods, χ^2^(1) = 192.28, *p* < .000. The original methodology achieved an overall 96.58% accuracy rate, whereas that of the revised methodology reached 92.74%. For the original method, only 8 individuals were incorrectly scored, while for the revised method 17 received an incorrect score. This translates to misclassification rates of 3.42% for the original and 7.26% for the revised method.

A McNemar’s test for the original method was used to determine whether there was a significant difference between males and females being sexed incorrectly. The sex accuracy of each sex for the original method can be found in Table 5.

**Table 5:** Distribution of sex accuracy for the Phenice method including sex bias

Accuracy of Sex	Male	Female	% Correct	% Incorrect	Sex Bias
Male	112	5	95.73%	4.27%	
(n = 117)					
Female	3	114	97.44%	2.56%	1.72%
(n = 117)					

The exact McNemar’s test determined there was no statistical significance between males and females being sexed incorrectly, *p* = .727. Only 3 females were incorrectly scored as male and 5 males were incorrectly scored as female. Overall, females were more likely to be sexed correctly, with an accuracy rate (97.44%) higher than that of males (95.73%), leading to a 1.71% sex bias in favour of females.

For the revised methodology, a chi-square test was run to determine whether there was a significant difference between males and females being sexed incorrectly. The sex accuracy of each sex for the revised methodology can be found in Table 6.

**Table 6:** Distribution of sex accuracy for the Klales et al. method including sex bias

Sex	Male	Female	Indeterminate	% Correct	% Incorrect	Sex Bias
Male	107	7	3	91.45%	8.55%	
(n = 117)						
Female	3	110	4	94.02%	5.98%	2.57%
(n = 117)						

The chi-square test demonstrated a statistically significant difference between males and females being sexed incorrectly, *χ*^*2*^(2) = 189.145*, p* < 0.000. Seven males were sexed incorrectly as females, and only 3 females were sexed incorrectly as males. This follows the pattern observed in the original methodology where males were more likely to be sexed incorrectly than females. The indeterminate scoring was not found to be statistically significant. Overall, for the revised methodology females were more likely to be scored correctly than males with accuracies of 94.02% and 91.45% respectively and a low sex bias towards females at 2.57%.

## Discussion

The focus of this study was to evaluate both the original and revised methodologies for sex assessment from the pubic bone on the same skeletal population [1, 2]. The results demonstrate that the application of the original method to the Lisbon population produced a higher accuracy rate, (96.58%) than the revised method (92.74%). Of the 234 skeletons from the Luís Lopes Anthropological Collection that were examined, only 8 individuals were sexed incorrectly when using Phenice’s original method, while 17 were sexed incorrectly for the revised. Out of the 234 innominates scored, the same five were sexed incorrectly by both the original and revised methodologies; possibly indicating that these particular specimens are outliers in their morphology. Fifty innominates were rescored and a high consistency for intra-observer scoring was found.

Overall, the accuracy of the original methodology in our results followed very closely the 96% that Phenice himself had reported [2]. Earlier studies that tested the validity of Phenice’s methodology had similar results with the original publication. This includes research by Schon, that also achieved an accuracy of 96% [23]. Even though Sutherland and Suchey’s main focus was the ventral arc, their test of the Phenice method also achieved an accuracy of 96% [24]. In a comparison of several macroscopic sex assessment techniques, Inskip and colleagues concluded that Phenice’s traits were most accurate, having a correct classification rate of 93.8% [15]. In 1978, Kelley’s study that had an accuracy of 90%, further validated the Phenice method [17]. Ubelaker and Volk reported equally good results with 88.4% [25]. Johnstone-Belford and colleagues achieved an overall accuracy of 92.24% when applying Phenice’s method to multi-detector computed tomography (MDCT) scans [16]. Barroso Flamino and colleagues used the original method on a sample of unknown sex; as a result their findings may not be reliable. However, they did state preference for Phenice’s original methodology on the basis of being more repeatable and less ambiguous [14]. Most recently, Oghenemavwe and Oludiniwa published a lower accuracy rate (81.82%) than what is reported in the literature for the original methodology [21]. The explanation offered by the authors to justify this lower rate was the overall younger age range of the sample and small sample size of just 27 individuals [21].

The reported results for the revised method in the present study appear to have a significantly greater accuracy rate (92.74%) when compared to the revised published rate (86.2%) [1]. When others attempted to replicate the revised methodology, including Klales on a different population, accuracy rates were generally higher than those of the 2012 publication [27, 28]. Gómez-Valdés and colleagues reported an overall accuracy of 94.6% on a modern Mexican population, which they then recalibrated to produce 100% accuracy [27]. Klales and Cole also checked the reliability of the revised methodology on a Hispanic sample, which achieved 90.3% accuracy, but only improved by 4% when they recalibrated it for the specific sample [28]. An additional study using the revised method was attempted on a global population; our results are most comparable to the White South African population at 94.0% rather than the U.S. White population at 90.7% [29]. Most recently, Selliah and colleagues reported an accuracy rate of 100% using the recalibrated regression formula of the revised methodology [30]. Further consideration should be noted in regards to the previous research utilizing the revised methodology mentioned here. All of the studies not only utilized the 1–5 ordinal scoring system, but also implemented the regression equation formulated in the 2012 publication [1, 27–30]. Notwithstanding, the purpose of the present study was to compare the visual criteria of both methods, not how well the regression equation fits the Lisbon population.

A secondary aspect of the research presented here was to determine whether there is a bias towards either of the sexes. It was found that there was no significant sex bias for the original method. Males were marginally more likely to be misclassified as females, with 5 males being scored incorrectly compared to 3 females. Overall, the accuracy rate for males was 95.73% and for females 97.44%. The current study’s results generally agree with those of Phenice [2]. Our accuracy rates are slightly higher for males than Phenice’s reported 95.62% and lower than his 100% accuracy for females. Inskip and colleague’s findings support a slight sex bias towards females with a 100% accuracy compared to 97.5% for males [15]. Johnstone-Belford and colleagues also reported similar findings, with 97.3% for females and 87.6% for males [16]. Ubelaker and Volk found females to be sexed correctly over males, but with a much higher sex bias; 97% for females compared to 79.8% for males [25].

The sex bias for the revised methodology was examined and in addition, as it contains a grading scale, there was a need to investigate whether one of the two sexes was more likely to be scored as indeterminate. Males were significantly more likely to be sexed incorrectly than females, with 7 males and 3 females being misclassified. There was no significant difference between males or females receiving an indeterminate score, with 3 males and 4 females placed in this category. Overall, females achieved a higher accuracy than males, with rates of 94.02% and 91.45%, respectively, which are both higher than those of Klales and colleagues [1]. Klales and colleagues found females to have a lower accuracy (85.75%) than males (90%), which is the opposite from our findings [1]. Other studies have also found that females have a higher accuracy rate than males [1, 27–30]. Gómez-Valdés and colleagues reported 100% accuracy for females compared to 86–92% for males [27]. Klales and Cole reported similar findings, with females at 96% and males 84.6% [28]. Kenyhercz and colleauges reported the same pattern found in our research, with females achieving higher accuracies than males [29]. Specifically, South African white and U.S. white females achieved 98% and 97.3% compared to 90% and 93.4% for their male counterparts [29]. In contrast to the current and previous findings, Selliah and colleagues (2020) reported 100% accuracy rates for both sexes.

The present study’s final interest was to determine whether any of the three traits were superior at sex determination. It was discovered that both methods had similar findings, with the highest accuracies for males found in the ventral arc (Tables 1, 2) which is also supported by previous research utilizing the original method [16, 17, 24]. However, there were two publications that found the subpubic concavity to have the highest accuracy in males [15, 21]. This is also supported by research utilizing the revised method [1, 27–29]. The subpubic concavity for females in this study is also found to have the highest accuracy for both methodologies which is supported by previous research [16, 21]. Our results differ from previous literature when utilizing the revised methodology. The presence of the ventral arc is reported as having the highest accuracy for females [1, 27–29]. A consistent report for both methodologies from previous studies is that of the medial aspect of the ischiopubic ramus that scores lowest when used alone [1, 2, 16, 17, 27–29]. This is consistent with our findings for females utilizing both methods but differs for males; when using the original method both the medial aspect of the ischiopubic ramus and subpubic concavity attained similar results and the subpubic concavity was the least accurate trait for the revised methodology.

The combined scores of both sexes for the original method correspond with previous reported patterns; the ventral arc having the highest accuracy, followed by the subpubic concavity and lastly the medial aspect of the ischiopubic ramus [2, 16, 17]. Although others have reported the subpubic concavity as having the highest accuracy rate [15, 21]. When sexes were combined for the revised method, the subpubic concavity had the highest accuracy rate, followed by the ventral arc and lastly the medial aspect of the ischiopubic ramus. These results differ from the Klales and colleagues publication where the ventral arc had the highest accuracy when used alone [1]. Not all studies have reported all the accuracy rates but instead identified a similar trend that was also found here, where the subpubic concavity and ventral arc are more reliable than the medial aspect of the ischiopubic ramus [28, 29].

The authors of the revised methodology justified the inclusion of intermediate scoring criteria due to every grade on the 1–5 ordinal scale being utilized within their research; while also found the original method lacking in capturing variation by not having one [1]. Despite the lack of intermediate scores in the original method, Phenice did acknowledge the chance of some ambiguity to be expected but not cause any serious problems, since it would be unlikely to occur in all three traits simultaneously [2]. In the present study, only 7 individuals were classified as ambiguous when utilizing the revised methodology, with the majority of the sample receiving a score of 1 (female) or 5 (male). Twenty-four received a score of 2 (probable female) and the same number received a score of 4 (probable male).

Since the 2012 revised publication, several researchers have tested the method proposed by Klales and colleagues. The present study is the first to compare the two methods on the same skeletal population, while also addressing the criticism made against the Phenice method. The original method does continue to hold ground within the field of biological anthropology, with its ability to be replicated over the past 50 years on various skeletal populations including modern ones. In our study both methods achieved high classification accuracies, with 100% observer accordance, but it was Phenice’s original method that was found to be superior at sex assessment. It should be noted again that the scope of this study was to test the visual scoring criteria of both the original Phenice and the 2012 revised methods and not how well the regression equation from the revised methodology fits the Lisbon population. Although the regression equation was not utilized for this particular study, it would be interesting for future research to explore how this compares to the original methodology and possibly develop a regression equation for it. Further investigation into potential age and ancestral related differences may aid in the refinement of the methodology and lead to consistently high accuracy rates across populations.

## Key points

1. The Phenice method is one of the most widely used sex assessment methods from the pubis.
2. The original Phenice method [2] and a recent revision by Klales et al. [1] were tested on the same skeletal population.
3. Results indicate that the original method performed better in a direct comparison with the revised methodology.
4. Both methods have high accuracy levels on the Portuguese population.
